# Mid-Ventricular Obliteration Diagnosed by Bedside-Focused Echocardiography in the Emergency Department in a Patient With Syncope and Associated Hypertrophic Cardiomyopathy: A Case Report

**DOI:** 10.7759/cureus.65066

**Published:** 2024-07-21

**Authors:** Jeremy J Webb

**Affiliations:** 1 Emergency Medicine, LewisGale Medical Center, Salem, USA

**Keywords:** mid-ventricular obliteration, spectral doppler, focused echocardiography, point-of-care ultrasound, syncope

## Abstract

Syncope is a common complaint encountered in emergency medicine practice with multiple potential etiologies to investigate. The utility of focused bedside echocardiography allows emergency physicians to diagnose acute cardiovascular causes in a time-sensitive fashion. In this case report, a 61-year-old female with mixed aortic valve disease presented to the emergency department after a syncopal episode. In addition to standard electrocardiogram and laboratory testing, investigation with focused echocardiography performed at the point of care revealed mid-ventricular obliteration due to hypertrophic myocardial remodeling, left ventricular underfilling, and hyperdynamic performance. Key echocardiographic findings that may assist emergency physicians in recognizing and managing this physiological entity were discussed.

## Introduction

Syncope is a frequently encountered chief complaint in the emergency department (ED), accounting for up to one million patient visits per year [1]. Cardiac etiologies include arrhythmia, ischemia, tamponade, valvular, and structural disease. Although such disease may present in isolation, it clinically may occur or be exacerbated by a combination of other variables including cardiac loading conditions, prescribed medication effects, and neuro-hormonal activation. Standard workup, including electrocardiogram (ECG), chest radiograph, and laboratory testing, may miss some of the above etiologies and combinations. The use of point-of-care ultrasound technology and the performance of a focused echocardiographic examination can assist the emergency medicine (EM) physician in the rapid diagnosis and initiation of management in such cases.

## Case presentation

Presenting concerns and clinical findings

A 61-year-old female presented to the emergency department (ED) with a chief complaint of syncope. Her medical history was significant for myocardial infarction with coronary stenting, ischemic stroke, hypertension, diabetes mellitus, and mixed aortic disease with moderate aortic stenosis and aortic regurgitation.

The patient was standing in her bathroom, having difficulty removing a lower denture, when she became lightheaded and suddenly lost consciousness. She awoke on the floor, and when she attempted to stand up, she suffered a second episode. She reported chronic exertional dyspnea, which mildly increased within the last week. She denied chest pain, pleurisy, orthopnea, palpitations, and extremity edema. She reported intermittent nausea and frequent anorexia that started with semaglutide initiation in the last six months. This became so problematic that she stopped the medication several weeks before her ED presentation. Her oral intake, however, had not improved. She was also taking a thiazide diuretic.

The patient was asymptomatic on ED arrival. Triage vital signs were stable with a blood pressure of 168/94 mmHg and a heart rate of 71 beats per minute. She was afebrile, with a normal respiratory rate and a room air oxygen saturation of 98%. Her examination was significant for a moderate systolic murmur and dry mucous membranes. An ECG was obtained, which revealed a normal sinus rhythm without signs of ischemia, QT prolongation, bundle branch or fascicular blocks, or pre-excitation. Laboratory testing revealed a normal complete blood count and normal chemistries, including electrolytes, glucose, and high-sensitivity troponin-I tests (Table 1).

**Table 1:** Emergency department laboratory testing

Lab		Normal range	Patient’s value
Complete blood count	White blood cell	4.50-10.50 x 10^3^/uL	9.41 x 10^3^/uL
	Hemoglobin	4.50-10.50 x 10^3^/uL	14.9 x 10^3^/uL
	Hematocrit	37.0%-47.0%	44.1%
	Platelet count	130-385 x 10^3^/uL	188 x 10^3^/uL
Basic metabolic panel	Sodium	135-145 mmol/L	138 mmoL/L
	Potassium	3.5-5.1 mmol/L	3.9 mmoL/L
	Chloride	98-107 mmol/L	104 mmol/L
	Carbon dioxide	20-31 mmol/L	24 mmol/L
	Blood urea nitrogen	9-23 mg/dL	15 mg/dL
	Creatinine	0.55-1.02 mg/dL	1.30 mg/dL
	Glucose	74-106 mg/dL	87 mg/dL
Cardiac enzyme	High-sensitivity troponin-I	<34.11 ng/L	22.4 ng/L

At this point, the working diagnosis was venous pooling, orthostasis, and volume depletion, which could reduce cardiac output in the setting of mixed aortic valve disease. Other differentials were also considered, including arrhythmogenic syncope and worsening aortic valvular disease. We aimed to further investigate with a focused transthoracic echocardiogram.

Focused echocardiography

The echocardiogram revealed a hyperdynamic left ventricle (LV), with signs of left ventricular hypertrophy (LVH) and kissing walls in an “hourglass” shape during systole (Video 1).

**Video 1:** Apical four-chamber view showing an underfilled LV with an estimated EF of 75%-80% and kissing LV walls with an "hourglass" shape during systole LV: Left ventricle; EF: Ejection fraction.

Complete mid-cavity obliteration was noted at the papillary level on short-axis imaging (Video 2). We interrogated the aortic valve with continuous wave Doppler that found a peak velocity of 3.0 m/s, similar to prior values recorded for this patient, and remained in the moderate category. She also had an element of aortic regurgitation, which was unchanged from previous assessments. The inferior vena cava measured 1.6 cm in diameter and collapsed more than 50% with inspiration.

**Video 2:** Subxiphoid short-axis view showing LV mid-cavity obliteration during systole at the papillary muscles level LV: Left ventricle.

**Figure 1:**
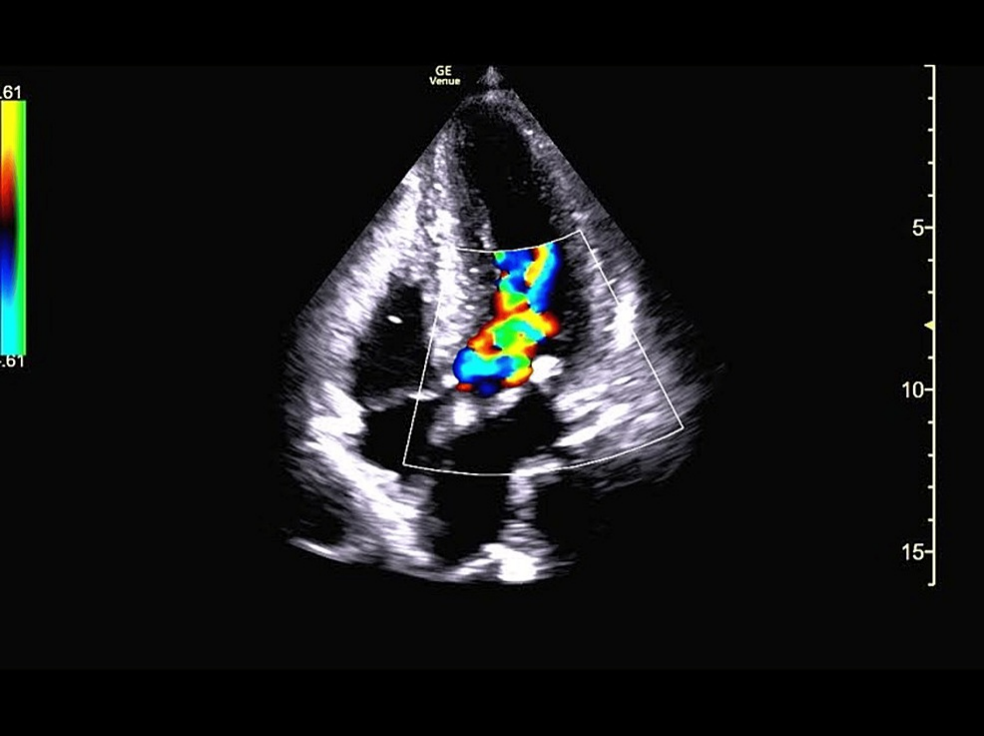
Apical four-chamber view revealing turbulence (aliasing with mixed color Doppler signal) within the LV outflow tract LV: Left ventricle.

Interrogation of the LV with color Doppler revealed turbulent flow (aliasing with mixed color Doppler signal) within the mid-ventricle and outflow tract (Figure 1).

**Figure 2:**
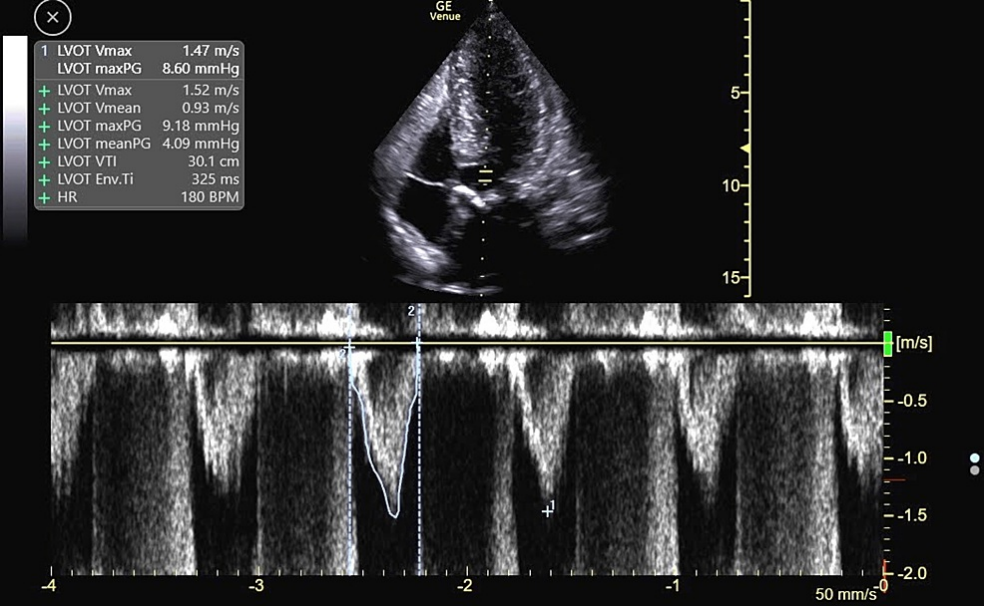
Pulsed-wave Doppler of the LV outflow tract revealing a late-peaking velocity envelope. Also, note the concomitant aortic regurgitation velocity profile during diastole. LV: Left ventricle.

**Figure 3:**
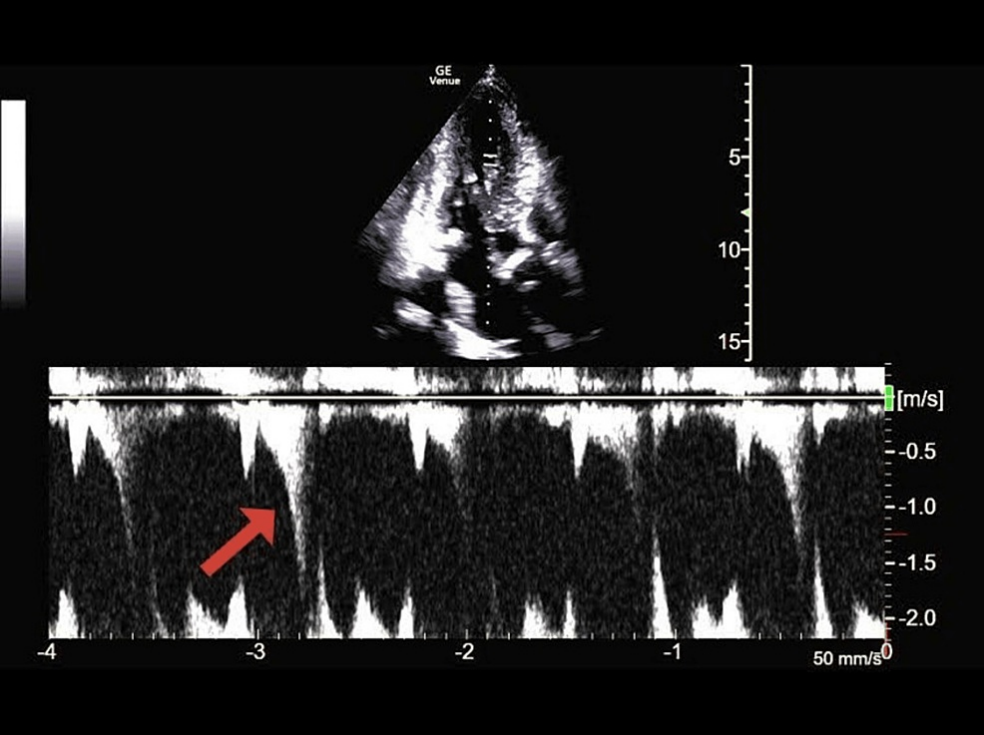
Pulsed-wave Doppler of the mid-LV cavity revealing a late-peaking, scythe-shaped velocity envelope LV: Left ventricle.

Using pulsed-wave Doppler, velocities from the LV outflow tract to the apex were sequentially sampled. This revealed a late-peaking LV outflow velocity profile that transitioned to a late-peaking, scythe-shaped velocity profile in the mid-ventricle (Figures 2, 3).

Patient course

Given the above, we suspected our patient suffered the effects of volume depletion and subsequently reduced preload while standing in her bathroom. This likely caused an underfilled LV, which created mid-cavity obliteration in the setting of LVH and a hyperdynamic LV. Coupled with mixed aortic disease, this likely further reduced cardiac output. Although provocative gradients were not obtained, suspicion of dynamic outflow obstructive physiology remained. Treatment in this case involved volume loading with intravenous fluid and continuation of beta-blocker therapy to avoid tachycardia. The patient was admitted for telemetry monitoring and was discharged from the hospital after a brief stay without further syncopal episodes.

## Discussion

Mid-ventricular obstruction is most often encountered in patients with hypertrophic cardiomyopathy [2]. However, additional etiologies such as LVH, sigmoid interventricular septum, and hypertrophied papillary muscles may also result in left ventricular cavity obliteration during systole and lead to an elevated mid-ventricular gradient [3-6]. Similar to hypertrophic cardiomyopathy, cases may be exacerbated with increased chronotropy/inotropy (e.g., exertion, dobutamine stress, inotropic medications, and endogenous catecholamine surge) or reduced preload (e.g., Valsalva, standing, over-diuresis, and volume depletion). Patients may present with exertional dyspnea or syncope and are at risk of ventricular arrhythmias [2,7]. It is also known that in patients with hypertrophic cardiomyopathy, mid-ventricular obstruction may be an independent predictor of adverse outcomes [2].

Obstructive sites to LV outflow are identified through sequential pulsed-wave Doppler waveform sampling from the LV outflow tract to the LV apex. The typical velocity profile is characterized by a late-peaking, scythe, or dagger-shaped envelope [8]. Occasionally, a mid-systolic drop may be detected, giving the profile a “lobster claw” appearance [9]. At rest, peak pressure gradients can be normal but may become significant (>30 mmHg) if provoked (e.g., Valsalva, squat to stand, and exercise/medication stress test).

The resting gradient in this patient was below 30 mmHg, and she was asymptomatic at the time of the exam. Provocative measurements were not obtained in the ED or inpatient setting. However, the Doppler envelope profile suggested dynamic outflow obstruction as a potential cause for this patient's syncopal event.

Advanced techniques, such as spectral Doppler physiological assessment, may require further continuing medical education and practice to become proficient in use, interpretation, and clinical application. However, focused echocardiography assessment in the ED setting can be performed with appropriate training and equipment, allowing EM clinicians to further investigate structural cardiac and hemodynamic issues in patients presenting with syncope.

## Conclusions

As this case illustrates, the use of bedside-focused echocardiography may assist EM physicians in navigating the differential diagnosis in presumed cardiogenic syncope and ruling out structural etiologies. It is a skill that can be learned and performed in the ED setting by EM physicians at the point of care. When assessing a patient with syncope, volume status and global cardiac function should be ascertained, especially in the setting of known valvular disease. The turbulent signal within the LV or outflow tract on color Doppler requires further investigation with pulsed-wave Doppler to evaluate for obstructive sites. Mid-ventricular obliteration may occur in the setting of hypertrophic cardiomyopathy or hypertensive heart disease and may precipitate syncopal events, especially when underfilled and hyperdynamic. Doppler envelope signals within the LV cavity or outflow tract that are late peaking or scythe-shaped may denote a dynamic obstruction. Gradients may be measured at rest and/or with provocation to identify significant physiological obstruction (>30 mmHg). If identified, initial treatment relies on adequate volume loading and avoidance of tachycardia.
